# Genomic, Antimicrobial, and Aphicidal Traits of *Bacillus velezensis* ATR2, and Its Biocontrol Potential against Ginger Rhizome Rot Disease Caused by *Bacillus pumilus*

**DOI:** 10.3390/microorganisms10010063

**Published:** 2021-12-29

**Authors:** Leiqin Liang, Yajuan Fu, Sangsang Deng, Yan Wu, Meiying Gao

**Affiliations:** 1 Wuhan Institute of Virology, Chinese Academy of Sciences, Wuhan 430071, China; liangleiqin@163.com (L.L.); fuyajuann@163.com (Y.F.); dengsangsang@163.com (S.D.); wuyan81@wh.iov.cn (Y.W.); 2 University of Chinese Academy of Sciences, Beijing 100039, China

**Keywords:** *Bacillus velezensis*, ginger rhizome rot disease, genome analysis, antimicrobial activity, aphicidal activity, bacillomycin D, biocontrol

## Abstract

Ginger rhizome rot disease, caused by the pathogen *Bacillus* *pumilus* GR8, could result in severe rot of ginger rhizomes and heavily threaten ginger production. In this study, we identified and characterized a new *Bacillus velezensis* strain, designated ATR2. Genome analysis revealed *B. velezensis* ATR2 harbored a series of genes closely related to promoting plant growth and triggering plant immunity. Meanwhile, ten gene clusters involved in the biosynthesis of various secondary metabolites (surfactin, bacillomycin, fengycin, bacillibactin, bacilysin, difficidin, macrolactin, bacillaene, plantazolicin, and amylocyclicin) and two clusters encoding a putative lipopeptide and a putative phosphonate which might be explored as novel bioactive compounds were also present in the ATR2 genome. Moreover, *B. velezensis* ATR2 showed excellent antagonistic activities against multiple plant pathogenic bacteria, plant pathogenic fungi, human pathogenic bacteria, and human pathogenic fungus. *B. velezensis* ATR2 was also efficacious in control of aphids. The antagonistic compound from *B. velezensis* ATR2 against *B.* *pumilus* GR8 was purified and identified as bacillomycin D. In addition, *B. velezensis* ATR2 exhibited excellent biocontrol efficacy against ginger rhizome rot disease on ginger slices. These findings showed the potential of further applications of *B. velezensis* ATR2 as a biocontrol agent in agricultural diseases and pests management.

## 1. Introduction

Ginger (*Zingiber officinale* Roscoe), a popular spice with aromatic and pungent components, is also utilized in traditional medicine for its healing properties of various gastrointestinal diseases, metabolic diseases, rheumatism, arthritis, pain, and muscle discomfort [1,2,3]. Moreover, numerous studies have reported that ginger exhibits antibacterial, antifungal, and anticancer properties [4,5,6]. Ginger rhizome is widely cultivated as an important economic crop. In ginger cultivation, agricultural diseases and pests are major factors restricting the production and quality of ginger [7,8,9,10,11]. Ginger rhizome rot disease, one of the most destructive diseases of ginger in Shandong Province of China, caused severe rot of ginger rhizomes during later growth stages and heavily threaten ginger production. The pathogen of ginger rhizome rot disease was identified as *B. pumilus* GR8 [12]. Currently, the control of ginger rhizome rot disease still primarily depends on preventive before-planting measures and agricultural chemicals.

In recent years, biological control agents (BCAs) with safe and eco-friendly merits have been regarded as an alternative strategy to chemical pesticides for controlling plant diseases and insect pests in the modern agricultural field [13]. Some *Bacillus* species, such as *B. subtilis*, *B. amyloliquefaciens*, and *B. licheniformis*, have been extensively studied and widely used to control various agricultural diseases and pests [14,15,16,17,18]. These *Bacillus* species harbor many advantages, such as high viability in different environmental conditions, harmlessness to plants and animals, low cost, and environmentally friendly.

*B. velezensis*, a *Bacillus* species first reported by Ruiz-García et al. in 2005 [19], was originally characterized as a later heterotypic synonym of *B. amyloliquefaciens* [20]. Recently, *B. methylotrophicus*, *B. oryzicola*, and *B. amyloliquefaciens* subsp. *plantarum* were reclassified as the later heterotypic synonyms of *B. velezensis* [21]. *B. velezensis* has many excellent merits, such as producing a variety of antimicrobial metabolites to suppress plant pathogens, enhancing plant defenses, and promoting plant growth and efficient colonization on plants [22,23,24,25]. Given these biocontrol properties, many strains of *B. velezensis* have been widely used in agricultural field [25,26,27]. For example, *B. velezensis* FZB42, a well-studied biocontrol strain, has been widely used as a disease suppressor and plant growth promoter for multiple economic crops and vegetables, such as lettuce, tobacco, cotton, tomato, and cucumber [23,28,29,30,31]. It was reported that *B. velezensis* CC09 exhibited strong antimicrobial activities and showed potential applications in control of wheat powdery mildew, take-all, and spot blotch diseases [13,32]. Furthermore, some *B. velezensis* strains encoded lignocellulolytic enzymes and exhibited the potential for degrading lignocellulosic biomass [33]. In addition, *B. velezensis* BZR 86 significantly controlled the root-knot disease on plants and noticeably increased growth and biomass of plants [34]. It was reported that bacterial treatment with *B. velezensis* YC7010 elicited the induced systemic resistance (ISR) and noticeably decreased settling, feeding, and reproduction of green peach aphid on *Arabidopsis* leaves [35]. However, few strains within *B. velezensis* have been reported with insecticidal activities against aphids.

In this study, we characterized a new *B. velezensis* strain, ATR2, which exhibited extensive antibacterial, antifungal, and aphicidal activities, identified the antagonistic compound from *B. velezensis* ATR2 against the pathogen of ginger rhizome rot disease, and evaluated the biocontrol efficacy of *B. velezensis* ATR2 against ginger rhizome rot disease. The results revealed the potential of further applications of *B. velezensis* ATR2 as a biocontrol agent in agricultural diseases and pest management.

## 2. Materials and Methods

### 2.1. Bacteria, Fungi, and Aphids Used in Assays

Strain ATR2 was originally isolated from the ginger rhizosphere in Shangdong Province (China) as the antagonistic strain against the pathogen of ginger rhizome rot disease (*B. pumilus* GR8). The pathogenic bacteria, *B. pumilus* GR8, *Yersinia pseudotuberculosis* YpⅢ, *B. anthracis*, *Klebsiella pneumoniae*, and *Acinetobacter baumannii* were stocked in our laboratory, and *Ralstonia solanacearum* (Smith), *Xanthomonas campestris* pv. *campestris*, *Enterobacter cloacae*, *Escherichia coli* EHEC O157:H7, *Shigella dysenteriae, Staphylococcus aureus* subsp. *aureus*, *Salmonella* Choleraesuis, and *Salmonella* Typhimurium were purchased from the BeNa Culture Collection (BNCC, Beijing, China).

The pathogenic fungi, *Fusarium graminearum* Sehw, *Botrytis cinerea*, *Ceratocystis paradoxa* (Dode) Moreau, and *Colletotrichum higginsianum* Sacc were kindly provided by Dr. Zhang from State Key Laboratory of Agricultural Microbiology, Huazhong Agricultural University, China, and *Microsporum gypseum* was purchased from the BNCC.

The aphids, *Uroleucon formosanum* Takahashi, *Aphis gossypii* Glover, *Aphis craccivora* Koch, and *Myzus percicae*, were obtained from local vegetable farms in Wuhan, Hubei, China.

### 2.2. Taxonomic Identification of Strain ATR2

To determine species identity of strain ATR2, bacterial cell and colony morphology, physiological and biochemical characteristics, 16S rRNA sequence, and complete genome sequence analyses were conducted [24,36]. Briefly, the features of cell morphology including shape, Gram staining, and spore forming were described using light microscopy (Olympus, Tokyo, Japan). The features of colony morphology were directly observed. The API 50 CHB strips were used to assess carbon source utilization and other physiological and biochemical properties of strain ATR2 according to the manufacturer’s instructions (bioMerieux, Marcy l’Etoile, France) [37].

For molecular identification, total bacterial DNA of strain ATR2 was extracted according to the protocol described by Gao et al. [38]. The 16S rRNA sequence was first amplified by polymerase chain reaction (PCR) using the primer pairs 27f/1492r with cycling conditions reported by Guardado-Valdivia et al. [39], detected with an agarose gel electrophoresis, purified with a Gel Extraction kit (Omega Bio-tek, Inc., Norcross, GA, USA), sequenced by TSINGKE Biological Technology Co., Ltd. (Beijing, China), and searched against the nucleotide sequences available in the National Center for Biotechnology Information (NCBI) nucleotide database. Based on 16S rRNA sequences of strain ATR2 and the type strains of other species from the NCBI nucleotide database, a phylogenetic tree was built using the neighbor-joining method with 1000 bootstrap replicates in MEGA software, version 7.0 [40]. Second, pairwise average nucleotide identity (ANI) values for genomes of strain ATR2 and 14 related type strains available in NCBI nucleotide database were calculated using JspeciesWS (http://jspecies.ribohost.com/jspeciesws/#home, accessed on 25 November 2019) [41]. A heatmap visualizing and hierarchal clustering the ANI values (%) was constructed using the online Morpheus software (https://software.broadinstitute.org/morpheus/, accessed on 24 August 2020).

### 2.3. Genome Sequencing, Assembly, Annotation, and Bioinformatics Analysis

The genomic DNA of strain ATR2 was sequenced on the Illumina HiSeq 2500 platform using a 500-bp paired-end library with read lengths of 150 bp. The raw reads were quality filtered by Quake [42]. The de novo assembly was performed by SPAdes 3.5.0 [43], and the resulting contigs longer than 500 bp were further arranged by comparing them with three reference genome sequences available in NCBI nucleotide database: *B. velezensis* YAU B9601-Y2 (GenBank accession no. NC_017061), *B. amyloliquefaciens* Y2 (GenBank accession no. NC_017912), and *B. subtilis* strain Bs-916 (GenBank accession no. NZ_CP009611) with Mauve (V2.4.0) to generate a draft genome with gaps. Finally, the gaps were bridged by high-fidelity PCR to acquire the complete genome. Genome annotation was conducted by NCBI Prokaryotic Genome Annotation Pipeline (PGAP, 2 March 2021, http://www.ncbi.nlm.nih.gov/genome/annotation_prok/) with best-placed reference protein set and GeneMarkS-2+ methods. Secondary metabolite clusters were identified by comparing ATR2 genome sequence with the antiSMASH database (11 May 2020, https://antismash.secondarymetabolites.org/) with default parameters [44]. The ATR2 genome sequence was also compared with the REBASE database (12 May 2020, http://rebase.neb.com/rebase/rebase.html) to predict the restriction modification (R-M) system [45]. The CRISPR/Cas (clustered regularly interspaced short palindromic repeats) system in the ATR2 genome was evaluated using the CRISPR finder online service (12 May 2020, https://crispr.i2bc.paris-saclay.fr/) [46]. The BLAST Ring Image Generator (BRIG) software was used to visualize the genome comparisons of strain ATR2 with four representative *Bacillus* strains: *B. subtilis* subsp. *subtilis* 168^T^ (GenBank accession no. AL009126), *B. subtilis* subsp. *inaquosorum* KCTC 13429^T^ (GenBank accession no. CP029465), *B. amyloliquefaciens* DSM 7^T^ (GenBank accession no. FN597644), and *B. velezensis* FZB42 (GenBank accession no. CP000560) [47].

### 2.4. UHPLC-QE-MS Analysis

For identifying the secondary metabolites synthesized by strain ATR2, an ultra-high performance liquid chromatography coupled with Q Exactive Orbitrap mass spectrometry (UHPLC-QE-MS) analysis was performed. Strain ATR2 was precultured overnight in 10 mL of Luria-Bertani (LB) liquid medium (tryptone 10 g/L, yeast extract 5 g/L, sodium chloride 10 g/L, the pH was adjusted to 7.0) at 28 °C and 180 rpm with a rotatory shaker (Zhicheng Industry Co., Ltd., Shanghai, China). An aliquot (2 mL) of the preculture (10^8^ CFU/mL) was added in a 500 mL shake flask containing 100 mL of YPD liquid medium (yeast extract 10 g/L, peptone 20 g/L, dextrose 20 g/L), and then incubated at 28 °C and 180 rpm with a rotatory shaker for 64 h. The cell free supernatant (CFS) was acquired by centrifugation of bacterial fermentation liquid at 4 °C and 8000 rpm for 10 min and filtrating the supernatant with a 0.22 μm filter (Merk Millipore Ltd., Tullagreen, Carrigtwohill, Co. Cork, Ireland).

The UHPLC separation conditions were as follows: a UPLC BEH Amide column (2.1 × 100 mm, 1.7 μm, Waters Corp., Milford, MA, USA) was attached to a 1290 Infinity series UHPLC System (Agilent Technologies Inc., Santa Clara, CA, USA), eluent A was 25 mM ammonium acetate and 25 mM ammonia hydroxide in water (pH = 9.75), eluent B was acetonitrile, flow rate was 0.5 mL/min, column temperature was 25 °C, injection volume was 3 μL, elution gradient was as follows: 0.0 min, 95% B; 0.5 min, 95% B; 7.0 min, 65% B; 8.0 min, 40% B; 9.0 min, 40% B; 9.1 min, 95% B; 12.0 min, 95% B. The Q Exactive Orbitrap mass spectrometer (Thermo Fisher Scientific Inc., Waltham, MA, USA) was used to obtain MS/MS spectra on information-dependent acquisition (IDA) mode using Xcalibur 4.0.27 software (Thermo Fisher Scientific Inc., Waltham, MA, USA) under the following conditions: sheath gas flow rate of 45 Arb, aux gas flow rate of 15 Arb, capillary temperature of 400 °C, full MS resolution of 70,000, mass range of 100–1500 *m*/*z*, MS/MS resolution of 17,500, collision energy of 10/30/60 in NCE mode, spray voltage of 4.0 kV (positive) or −3.6 kV (negative), respectively [48].

### 2.5. Antimicrobial Assays of Extracellular Metabolites from Strain ATR2

To evaluate the antagonistic effect of the extracellular metabolites on some pathogenic microorganisms, antimicrobial assays were performed with the CFS of strain ATR2. For the antibacterial activity detection, hole-plate diffusion assays were conducted as described by Njimoh et al. with minor modification [49]. Briefly, the pathogenic bacteria were cultured in LB liquid mediums at 37 °C except *Ralstonia solanacearum* in 950 liquid medium (peptone 5 g/L, dextrose 5 g/L, beef extract 3 g/L, yeast extract 0.25 g/L, sodium chloride 0.5 g/L, magnesium sulfate heptahydrate 0.5 g/L, the pH was adjusted to 7.0) at 28 °C using a rotatory shaker (180 rpm). Thereafter, 200 μL of the pathogenic bacterial culture (0.5 McFarland standard) was added into 20 mL of melted and tempered (50 °C) LB agar mediums except *Ralstonia solanacearum* was added into 950 agar medium which was mixed rapidly and poured in the sterile petri dishes (90 mm in diameter). After solidification of the medium at room temperature, three holes evenly distributed on the LB agar plate were created with a sterilized puncher (10 mm in diameter) and filled with 100 μL of the CFS. An equal volume of YPD liquid medium was used to serve as the blank control. After being kept at room temperature for 1 h, all the plates were cultivated at 37 °C for 24 h except *Ralstonia solanacearum* at 28 °C. The antibacterial activities were determined based on the presence of inhibition zones around the holes, and the diameters of inhibition zones were measured. The experiments were carried out three times.

The potential antifungal activities of extracellular metabolites from strain ATR2 were evaluated using the plate confrontation method described by Jiang et al. with minor modification [25]. Briefly, the pathogenic fungi were cultured on potato dextrose agar (PDA) plates (potato 200 g/L, dextrose 20 g/L, agar 15 g/L) at 28 °C for 5 days, then a mycelia disc was made with a sterilized puncher (10 mm in diameter) and inoculated at the center of a petri dish containing 25 mL of PDA medium. Four holes were punched at 20 mm from the center, and two holes on both sides of the plate were filled with 200 μL of the CFS. As a control, the other two holes were filled with the same volume of YPD liquid medium. The plates were cultured at 28 °C until the mycelial growth on the control sides was almost complete. The diameter of mycelial growth on treated sides (*T*) and the diameter of mycelial growth on control sides (*C*) were measured and the inhibition ratio of fungal growth (*I*) was calculated by the formula: *I*% = ((*C* − *T*)/(*C* − 10)) × 100 [24,39]. Tests were repeated three times.

### 2.6. Aphicidal Assays of Extracellular Metabolites from Strain ATR2

The aphicidal activities of extracellular metabolites from strain ATR2 were evaluated using leaf-disc spraying method according to the guideline for laboratory bioassay of pesticides. Briefly, the leaves of host plants with uniform growth were divided into small pieces (60 × 30 mm) and placed in petri dishes (90 mm in diameter). Thirty aphids with the same physiological state were picked from the local vegetable farms and allowed to locate on a leaf for 3 h prior to application [16]. Then, 2 mL of the fermentation supernatant diluent (10% dilution, *v*/*v*) was evenly sprayed on the aphids with a small sprayer. After the fermentation supernatant diluent settled for 1 min, the leaf was transferred to a new petri dish which was layered with a piece of wet filter paper and punched with a needle to make some air vents. The same volume of PBS buffer (KH_2_PO_4_ 0.24 g/L, Na_2_HPO_4_ 1.42 g/L, NaCl 8 g/L, KCl 0.2 g/L, the pH was adjusted to 7.4) was used as the blank control. The dishes were kept in a growth chamber at 25 °C, a relative humidity of 40 to 70% and a photoperiod of 16:8 h (L:D) for 48 h after application. When the mortality of the control group was less than 10%, the corrected mortality was calculated by Abbott’s formula [50]: corrected mortality (%) = ((*A* − *B*)/*A*) × 100, where *A* and *B* represented the number of the living aphids in the control group and the number of the living aphids in the treated group, respectively. The experiments were repeated three times.

To assess the aphicidal activities in the field, the fermentation supernatant of strain ATR2 was sprayed evenly on stems and leaves of the vegetables severely attacked by aphids. An equal volume of PBS buffer was served as the blank control. The control efficacy was assessed at 24 h after application. The experiments were repeated three times when the weather was sunny, the temperature was between 15 °C and 25 °C, and the wind speed was level 2 to level 3.

### 2.7. Purification and Mass Spectrometry Analysis of the Antagonistic Compound against B. pumilus GR8

For identifying the antagonistic compound from strain ATR2 against *B. pumilus* GR8, crude antibacterial substances were first obtained following the extraction method described by Gong et al. with minor modification [51]. Briefly, the fermentation supernatant of strain ATR2 was treated with 4 M HCl to adjust pH to 2.0 and then kept at 4 °C overnight. When the supernatant after acid precipitate had no antagonistic activity against *B. pumilus* GR8, the precipitation was obtained by centrifugation at 8000 rpm, 4 °C for 20 min and solubilized in a minimal volume of PBS buffer with 10 M NaOH to pH 7.5. After recentrifugation at 8000 rpm for 15 min, the crude antibacterial substances were thus acquired in the soluble fraction. Then, the crude solution was purified by preparative thin layer chromatography (TLC) on silica gel GF254 plates (1 mm, Xinchu Industry Co., Ltd., Shanghai, China) developed with a methanol: acetone (9:1, *v*/*v*) solvent system. Each fraction of the plate was extracted with methanol, and then the methanol extract was evaporated to dryness under vacuum with a rotary evaporator, solubilized in PBS buffer, and tested for its antagonistic activity against *B. pumilus* GR8. The active fraction was further purified by a semi-preparative HPLC system (1200 series, Agilent Technologies Inc., Santa Clara, CA, USA) using a Polar-HILIC column (10.0 × 250 mm, 5 μm, Sepax Technologies, Inc., Newark, DE, USA) eluting with a solvent containing 60% A (HPLC grade methanol) and 40% B (5 mM ammonium acetate in HPLC grade water, pH 7.5) at a flow rate of 1 mL/min. Elution was monitored by an ultraviolet (UV) detector (Agilent Technologies Inc., Santa Clara, CA, USA) at 220 nm.

A liquid chromatography coupled with electron spray ionization tandem mass spectrometry (LC–ESI–MS/MS) system (Ultimate 3000 LCQ FLEET, Thermo Fisher Scientific Inc., Waltham, MA, USA) was employed for the identification of accurate molecular weight and molecular formula of the antagonistic compound from strain ATR2 against *B. pumilus* GR8. The elution solvent was the same as the semi-preparative HPLC above at a flow rate of 0.3 mL/min, and the program was supervised by UV detection at 254 nm. The MS analysis was performed at a spray voltage of 4.0 kV for positive mode, a capillary temperature of 300 °C and a mass range of 100–2000 *m*/*z*.

### 2.8. Biocontrol Assays of Strain ATR2 against Ginger Rhizome Rot Disease

To evaluate the biocontrol efficacy of strain ATR2 against ginger rhizome rot disease, biocontrol assays were performed on ginger rhizome slices as described by Ye et al. with minor modification [52]. Briefly, antagonistic bacterium strain ATR2 and pathogenic bacterium *B. pumilus* GR8 were cultivated in YPD liquid medium and LB liquid medium at 28 °C and 180 rpm with a rotatory shaker for 16 h, respectively. Ginger rhizomes purchased from a market were washed in tap water and dried at room temperature. Surface disinfection was conducted by immersing ginger rhizomes sequentially in 70% ethanol and sterile distilled water (SDW) for 1 min followed by air-drying at room temperature for 20 min. After the disinfection procedures were repeated three times, ginger rhizome slices 3 mm in thickness were prepared with a flame-sterilized knife. A 100-μL bacterial suspension of GR8 (2 × 10^8^ CFU/mL) and a 100-μL bacterial suspension of ATR2 at approximately 2 × 10^9^, 2 × 10^8^, or 2 × 10^7^ CFU/mL were mixed and inoculated into the center of ginger rhizome slices. After inoculation, the slices were placed in sterile petri dishes and incubated at 30 °C and 40 to 70% relative humidity for about 2 days. Three control groups included 200-μL bacterial suspension of ATR2 (10^9^ CFU/mL), 200-μL bacterial suspension of GR8 (10^8^ CFU/mL), and 200-μL YPD liquid medium. The symptom severity of the ginger slices was assessed as described by Nishijima et al. [53]. Ginger slices were rated using a scale of 1 to 5 according to the percentage of rot region, where 1 = healthy tissue or tissue without symptoms, 2 = slight (1 to 25%) rot (slight discoloration), 3 = moderate (26 to 50%) rot (discoloration and tissue breakdown), 4 = severe rot (51 to 75%), and 5 = complete rot (76 to 100%). The trials were performed in triplicate.

### 2.9. Accession Numbers

The 16S rRNA sequence and complete genome sequence of strain ATR2 have been deposited in GenBank under the accession number KP685408 and NZ_CP018133, respectively. Strain ATR2 is available in China Center for Type Culture Collection (CCTCC NO. M2016071).

## 3. Results

### 3.1. Strain Identification

**Figure 1:**
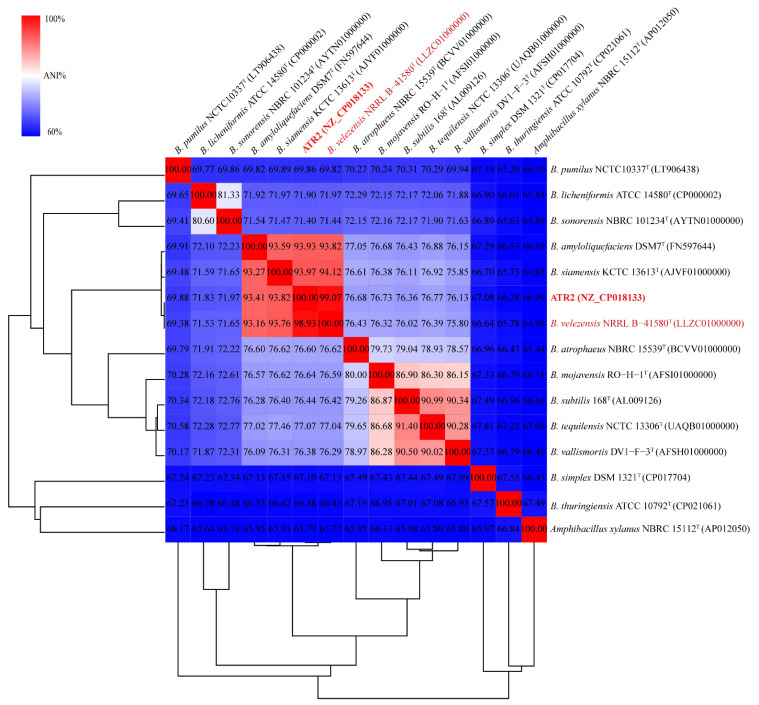
Heat-map analysis based on the ANI values of strain ATR2 and related strains. Strain ATR2 was highlighted in bold with red letters. *B. velezensis* NRRL B-41580^T^ was highlighted with red letters. Numbers in parentheses represented the GenBank accession numbers.

Morphological observation revealed the cells of strain ATR2 were Gram-positive, with spores and rod-shaped (Figure S1A). Strain ATR2 generally formed creamy white, opaque, rough colonies with irregular edge on LB agar medium. The colony morphology of strain ATR2 was quite different from that of *B. velezensis* FZB42, *B. amyloliquefaciens* DSM 7^T^, and *B. subtilis* 168^T^ (Figure S1B). Analysis of physiological and biochemical characteristics showed strain ATR2 was positive for starch hydrolysis and oxidase reaction, but negative for anaerobic growth. Meanwhile, strain ATR2 utilized L-arabinose, D-ribose, D-xylose, sorbitol, methyl-α-D-glucoside, D-lactose, D-melibiose, D-trehalose, D-raffinose, glycogen, and D-turanose (Table S1). Based on the 16S rRNA sequences analysis, strain ATR2 (GenBank accession KP685408) exhibited 98.46% sequence identity with *B. velezensis* strain EH9 (GenBank accession MN750764) and *B. velezensis* strain H34 (GenBank accession KJ149808). The phylogenetic analysis of 16S rRNA sequences showed strain ATR2 also clustered with *B. velezensis* (Figure S2). Furthermore, strain ATR2 shared 99.07% identity with the type *B. velezensis* strain NRRL B-41580 according to ANI values calculation. It could be considered as *B. velezensis* based on the generally accepted species boundary of 95% cut-off ANI value [41,54] (Figure 1), in accordance with the result of 16S rRNA sequences analysis. Therefore, strain ATR2 was identified as a member of *B. velezensis* based on cell and colony morphology, physiological and biochemical characteristics, phylogenetic taxonomy, and ANI values.

### 3.2. Genomic Features of B. velezensis ATR2

Based on the genome sequencing and assembly, a total of 9,779,334 reads were obtained and assembled into 40 contigs with a sequencing depth of 294-fold. The gaps between contigs were bridged by high-fidelity PCR. The complete genome of *B. velezensis* ATR2 was 4,006,746 bp, containing a circular chromosome with 46.34% of G + C content, and possessed 3745 protein-coding genes, 21 rRNA genes, and 69 tRNA genes. Comparative genomic analysis revealed that the genome of *B. velezensis* ATR2 showed 90.69% similarity to the genome of *B. velezensis* FZB42 (Figure S3), which had been widely used as biocontrol agent and biofertilizer. The R-M system and CRISPR/Cas system were two main defense systems that widely existed in the prokaryotic organisms to protect the bacteria from the foreign DNA. Only one type IV R-M system was identified in the ATR2 genome by comparing with the REBASE database. The type IV R-M system was composed of one or two genes encoding restriction enzymes that cleaved only modified DNA, including methylated, hydroxymethylated, and glucosyl-hydroxymethylated bases. Moreover, only a questionable CRISPR/Cas system was detected in the ATR2 genome using the CRISPR finder online service, which had only two direct repeats (DRs) and the repeated motifs (DR in CRISPR) were not 100% identical. The lack of strict defense system in the ATR2 genome laid a promising foundation for genetic manipulation of *B. velezensis* ATR2.

### 3.3. Genes Involved in Plant Growth Promotion

Considering that some biocontrol agents not only prevented plant disease but also participated in plant growth promotion, we also analyzed the genes involved in plant growth promotion in the genome of *B. velezensis* ATR2. As shown in Table 1, *B. velezensis* ATR2 harbored a series of genes associated with nutrition utilization and production of multiple growth-promoting substances, including 3-hydroxy-2-butanone, 2,3-butanediol, indole acetic acid (IAA), phytase, trehalose, spermidine, and bacillibactin. The *dhb* gene cluster was responsible for encoding the synthetase of the iron-siderophore bacillibactin which absorbed and accumulated iron ions from the environment under iron limitation. The 3-hydroxy-2-butanone and 2,3-butanediol, two well-known volatile organic compounds (VOC), exhibited large capacity to promote plant growth and elicit the ISR against plant pathogens. Genes involved in the synthesis of the two growth-promoting VOCs including acetolactate synthase small subunit (*ilvN*), acetolactate synthase large subunit (*ilvB*), alpha-acetolactate decarboxylase (*alsD*), acetolactate synthase (*alsS*), and butanediol dehydrogenase (*bdhA*) were present in the ATR2 genome. Meanwhile, genes associated with the synthesis of growth-promoting hormones, including indole-3-glycerol phosphate synthase (*trpC*), 3-phytase (*phyC*), trehalose permease IIC protein (*treP*) and trehalose operon repressor (*treR*), were also found in the ATR2 genome. Indole acetic acid (IAA), a well-characterized phytohormone, effectively promoted plant growth and its biosynthesis significantly affected the plant growth-promoting activity. The phytase, a functional enzyme degrading the extracellular phytate, was important for the plant growth-promoting effect under phosphate limitation. Trehalose, a nonreducing disaccharide, was accumulated by bacteria as a strategy for protecting cells from desiccation and water loss. In addition, genes responsible to produce spermidine, including agmatinase (*speB*) and spermidine synthase (*speE*), were discovered in the ATR2 genome. Spermidine, a type of polyamine (PA), played an important role in improving plant growth and drought tolerance. Genes related to the transport and utilization of nitrogen, potassium, and magnesium were summarized in Table 1.

### 3.4. Genes Involved in Secondary Metabolite Synthesis

As we all know, some biocontrol *Bacillus* species exhibit powerful antimicrobial capacities as a result of producing kinds of bioactive metabolites. A total of twelve gene clusters associated with secondary metabolite synthesis were found in the ATR2 genome. Among them, three giant gene clusters (*mln, bae*, and *dif*), covering altogether over 196 kb, were predicted to synthesize three polyketides (macrolactin, bacillaene, and difficidin). Moreover, five gene clusters (*srf*, *bmy*, *fen*, *bac*, and *dhb*) participated in nonribosomal synthesis of three cyclic lipopeptides (surfactin, bacillomycin, and fengycin), one dipeptide (bacilysin), and one siderophore (bacillibactin). In addition, two gene clusters (*acn* and *ptn*) involved in ribosomal synthesis of one bacteriocin (amylocyclicin) and one modified peptide (plantazolicin) were also found in the ATR2 genome (Figure S3, Table 2).

**Figure 2:**
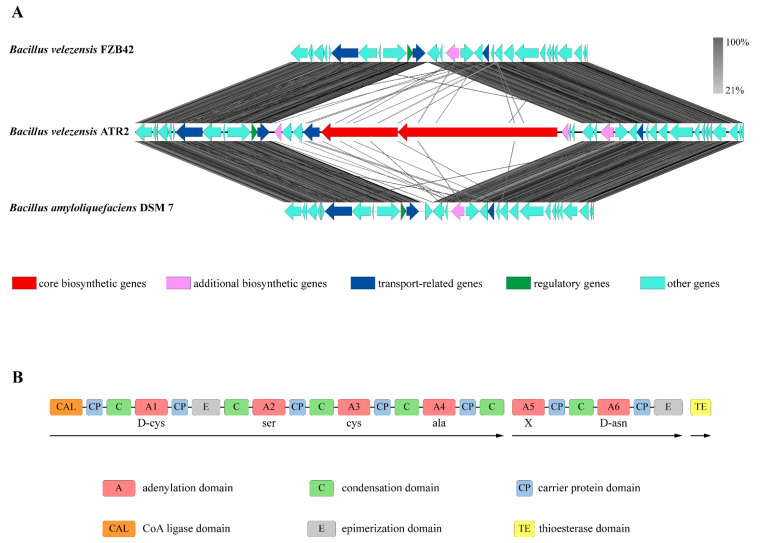
Gene cluster for the putative lipopeptide. (**A**), The putative lipopeptide gene cluster and surrounding genes on the *B. velezensis* ATR2 chromosome. The same locus of *B. velezensis* FZB42 and *B. amyloquefaciens* DSM 7^T^ were also presented for comparison. Gray bars indicated the similarity which was calculated via tblastx with the Easyfig tool [58]. (**B**), Schematic representation of the putative lipopeptide gene cluster. Three NRPS subunits were indicated with arrows. The predicted amino acids were also shown (X for unidentified amino acid). In addition, functional protein domains were indicated as A for adenylation domain; C, condensation domain; CP, carrier protein domain; E, epimerization domain; CAL, CoA ligase domain; and TE, thioesterase domain.

In addition to ten gene clusters associated with the known bioactive metabolites, the other two clusters encoding a putative lipopeptide and a putative phosphonate were also detected in the ATR2 genome which were not found in the genomes of *B. velezensis* FZB42, *B. amyloliquefaciens* DSM 7^T^, and *B. subtilis* 168^T^ (Figure S3, Table 2). Interestingly, genes surrounding the gene cluster encoding the putative lipopeptide on *B. velezensis* ATR2 chromosome shared high degrees of homology with the same locus of *B. velezensis* FZB42 and *B. amyloquefaciens* DSM 7^T^. Figure 2A suggested that the gene cluster encoding the putative lipopeptide might insert into *B. velezensis* ATR2 chromosome as a single copy. The gene cluster should encode a hexapeptide when the biosynthesis obeyed the linear mechanism. Meanwhile, the amino acid sequence of the putative lipopeptide was determined by the specificity and order of the hexamodular NRPSs. However, the hexamodular NRPSs might also encode other lipopeptide when the biosynthesis deviated from the linear rule and presented a nonlinear assembly like locillomycins [55]. Moreover, the putative lipopeptide was predicted to contain two cysteine residues (Figure 2B) which were the signatures for some antibacterial and antifungal polypeptides with cysteine-knot structural motifs [56,57].

**Figure 3:**
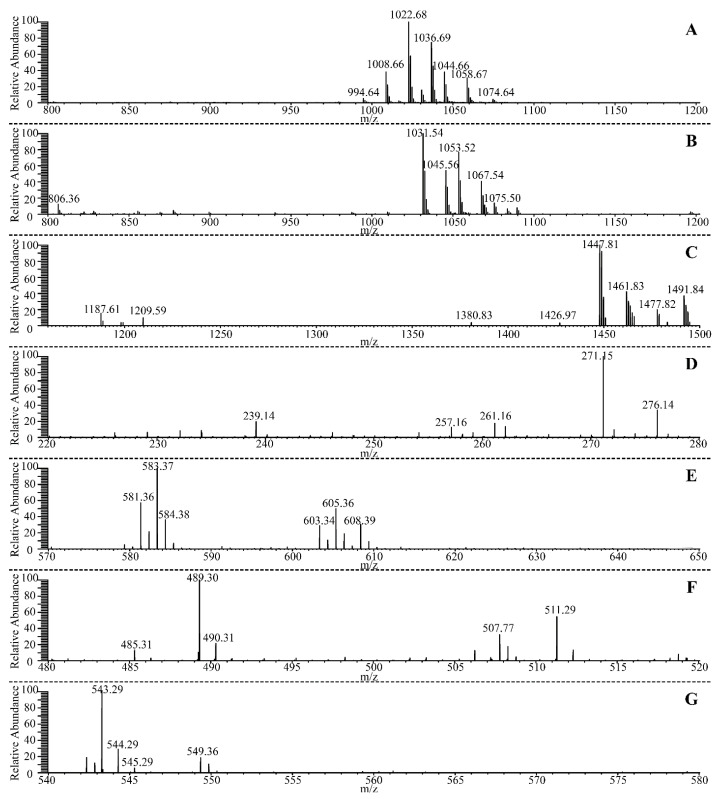
UHPLC-QE-MS analysis of secondary metabolites synthesized by *B. velezensis* ATR2. (**A**), Ions of *m*/*z* 994.64, 1008.66, 1022.68, and 1036.69 corresponded to the surfactin A (M + H)^+^, and ions of *m*/*z* 1044.66 and 1058.67 corresponded to surfactin A (M + Na)^+^; (**B**), ions of *m*/*z* 1031.54 and 1045.56 corresponded to bacillomycin D (M + H)^+^, and ions of *m*/*z* 1053.52 and 1067.54 corresponded to bacillomycin D (M + Na)^+^; (**C**), ions of *m/z* 1447.81, 1461.83, 1477.82, and 1491.84 corresponded to fengycin (M + H)^+^; (**D**), ion of *m*/*z* 271.15 corresponded to bacilysin (M + H)^+^; (**E**), ions of *m*/*z* 581.36 and 583.37 corresponded to bacillaene A (M + H)^+^ and bacillaene B (M + H)^+^, respectively, and ions of *m*/*z* 603.34 and 605.36 corresponded to bacillaene A (M + Na)^+^ and bacillaene B (M + Na)^+^, respectively; (**F**), ion of *m*/*z* 489.30 corresponded to 7-*O*-malonyl macrolactin A (M + H)^+^, and ion of *m*/*z* 511.29 corresponded to 7-*O*-malonyl macrolactin A (M + Na)^+^; (**G**), Ion of *m*/*z* 543.29 corresponded to difficidin (M − H)^−^.

Furthermore, three cyclic lipopeptides (surfactin, bacillomycin D, and fengycin), three polyketides (macrolactin, difficidin, and bacillaene) and one dipeptide (bacilysin) were detected by UHPLC-QE-MS analysis (Figure 3), confirming their successful production from *B. velezensis* ATR2. Analysis of the secondary metabolites synthesized by *B. velezensis* ATR2 offered an effective way to understand the mechanisms of its multiple bioactivities and facilitated the exploitation for novel bioactive metabolites.

### 3.5. Antimicrobial Activities of Extracellular Metabolites from B. velezensis ATR2

The antimicrobial activities of extracellular metabolites from *B. velezensis* ATR2 were measured by hole-plate diffusion assays for pathogenic bacteria and plate confrontation assays for pathogenic fungi. The results of antibacterial assays indicated that *B. velezensis* ATR2 displayed broad antibacterial spectrum and strong bacteriostatic activities against multiple plant pathogenic bacteria, e.g., *Bacillus pumilus* GR8, *Ralstonia solanacearum* (Smith), *Xanthomonas campestris* pv. *campestris*, and *Enterobacter cloacae*, and human pathogenic bacteria, e.g., *Yersinia pseudotuberculosis* YpⅢ, *Bacillus anthracis*, *Escherichia coli* EHEC O157:H7, *Klebsiella pneumoniae*, *Shigella dysenteriae*, *Staphylococcus aureus* subsp. *aureus*, *Salmonella* Choleraesuis, *Salmonella* Typhimurium, and *Acinetobacter baumannii* (Table 3). Furthermore, *B. velezensis* ATR2 also significantly inhibited the mycelia growth of *Fusarium graminearum* Sehw, *Botrytis cinerea*, *Ceratocystis paradoxa* (Dode) Moreau, *Colletotrichum higginsianum* Sacc, and *Microsporum gypseum*, with 75.42%, 80.83%, 91.67%, 81.67%, and 52.5% of inhibition ratio, respectively (Table 3).

### 3.6. Aphicidal Activities of Extracellular Metabolites from B. velezensis ATR2

**Figure 4:**
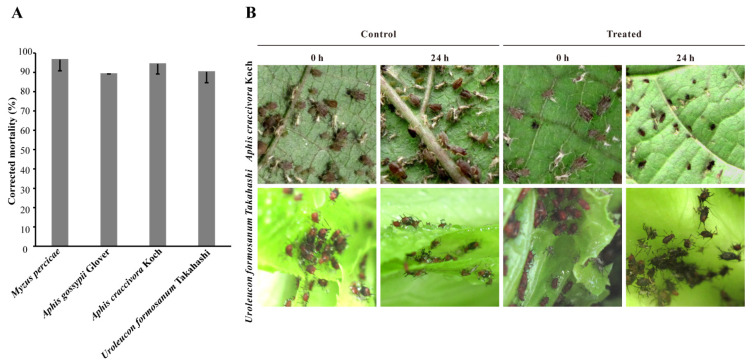
Insecticidal activity of the fermentation supernatant from *B. velezensis* ATR2. (**A**), Aphicidal activity of the fermentation supernatant diluent (10% dilution) against the tested aphids in laboratory bioassays. (**B**), Aphicidal activity of the fermentation supernatant against *Aphis craccivora* Koch and *Uroleucon formosanum* Takahashi in the field. Means and standard deviations were statistically calculated using the software package SPSS v18.0.

The aphicidal activities of extracellular metabolites from *B. velezensis* ATR2 were assessed according to the guideline for laboratory bioassay of pesticides, and the results revealed that the fermentation supernatant diluent (10% dilution, *v*/*v*) exhibited excellent aphicidal activities on *Uroleucon formosanum* Takahashi, *Aphis gossypii* Glover, *Aphis craccivora* Koch, and *Myzus percicae*, with 90.24%, 89.15%, 94.32%, and 96.63% of corrected mortality, respectively (Figure 4A). The aphicidal activities of *B. velezensis* ATR2 in the field were observed at 0 h and 24 h after application, respectively. The results indicated that the fermentation supernatant of *B. velezensis* ATR2 showed a strong toxic effect on bean aphids (*Aphis craccivora* Koch) and the aphids on the leaves almost died at 24 h after application, and the dead insect body shriveled and blackened. The same toxic effect was also exhibited on lettuce aphids (*Uroleucon formosanum* Takahashi) at 24 h after application (Figure 4B). These results indicated that *B. velezensis* ATR2 was able to effectively control aphids as a biological pesticide.

### 3.7. Identification of the Antagonistic Compound against B. pumilus GR8

**Figure 5:**
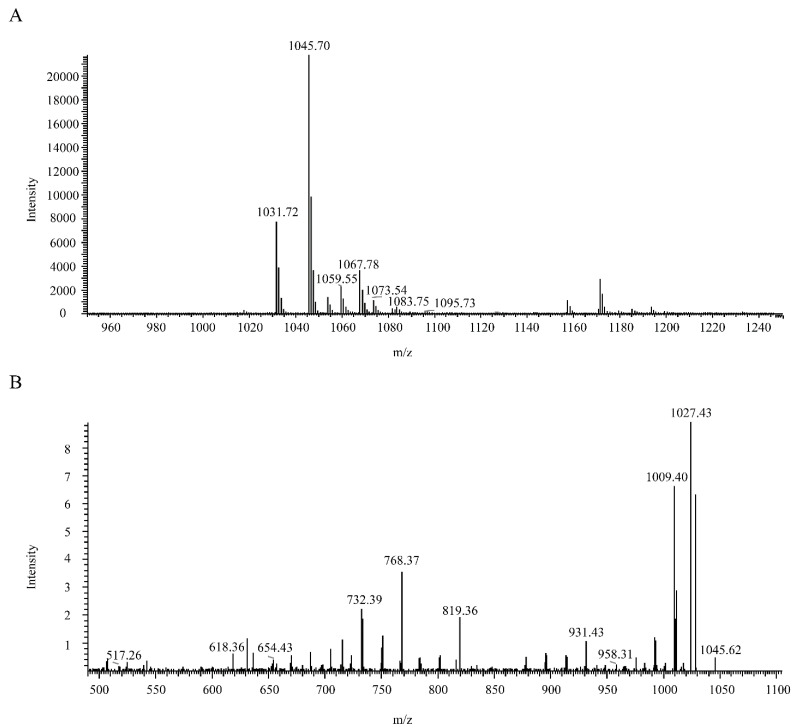
HPLC-ESI-MS analysis of the purified antagonistic compound from *B. velezensis* ATR2 against *B. pumilus* GR8 (**A**) and CID spectrum resulting from (M + H)^+^ ion of *m*/*z* 1045.70 (**B**). (**A**), Ions of *m*/*z* 1031.72, 1045.70, 1059.55 and 1073.54 corresponded to bacillomycin D (M + H)^+^, ions of *m*/*z* 1067.78 and 1095.73 corresponded to bacillomycin D (M + Na)^+^, and ion of *m/z* 1083.75 corresponded to bacillomycin D (M + K)^+^; (**B**), ion of *m*/*z* 1027.43 corresponded to (M + H − H_2_O)^+^, ion of *m*/*z* 958.31 corresponded to (M + H − Ser)^+^, ion of *m*/*z* 931.43 corresponded to (M + H − Asn)^+^, ion of *m*/*z* 819.36 corresponded to (M + H − Pro − Glu)^+^, ion of *m*/*z* 768.37 corresponded to (M + H − Tyr − Asn)^+^, ion of *m*/*z* 732.39 corresponded to (M + H − Pro − Glu − Ser)^+^, ion of *m*/*z* 654.43 corresponded to (M + H − Asn − Tyr − Asn)^+^, ion of *m*/*z* 618.36 corresponded to (M + H − Asn − Pro − Glu − Ser)^+^, and ion of *m*/*z* 517.26 corresponded to (M + H − Asn − Pro − Glu − Ser − Thr)^+^.

By acid precipitate from the fermentation supernatant of *B. velezensis* ATR2, the crude antibacterial substances were obtained, and most of the impurities were excluded. With preparative TLC and subsequent semi-preparative HPLC, a peak with antagonistic activity was purified (Figure S4). Therefore, the active peak was subjected to LC–ESI–MS/MS for its molecular weight and molecular formula identification. The data from mass spectrum analysis in the positive-ion mode revealed that (M + H) ^+^ ions were at 1031.72, 1045.70, 1059.55, and 1073.54 *m*/*z*, respectively (Figure 5A). The interval of 14 mass unit was a common phenomenon for molecular weight of many cyclic lipopeptides secreted by *Bacillus* strains as a result of different numbers of methylene units in lipid chains. Based on the mass data in published studies [75,76], the active peak may be recognized as the homologues of bacillomycin D with molecular weights of 1030 Da (C14), 1044 Da (C15), 1058 Da (C16), and 1072 Da (C17).

ESI/CID-MS spectrometry analysis of precursor ion of *m*/*z* 1045.70 was performed to further confirm the molecular formula of the antagonistic compound against *B. pumilus* GR8. The product ion of *m*/*z* 1027.43 in the CID spectrum could be elucidated as (1045.70 − 18) ^+^ which was identical to (M + H − H_2_O) ^+^. Similarly, product ions of *m*/*z* 958.31, 931.43, 819.36, 768.37, 732.39, 654.43, 618.36, and 517.26 in the CID spectrum presented (M + H − Ser) ^+^, (M + H − Asn) ^+^, (M + H − Pro − Glu) ^+^, (M + H − Tyr − Asn) ^+^, (M + H − Pro − Glu − Ser) ^+^, (M + H − Asn − Tyr − Asn) ^+^, (M + H − Asn − Pro − Glu − Ser) ^+^, and (M + H − Asn − Pro − Glu − Ser − Thr) ^+^, respectively (Figure 5B). Therefore, the amino acid composition of the antagonistic compound was (Asn-Tyr-Asn-Pro-Glu-Ser-Thr), which was identical to the amino acid residue of bacillomycin D [51,77]. Collectively, the antagonistic compound from *B. velezensis* ATR2 against *B. pumilus* GR8 was identified as bacillomycin D.

### 3.8. Biocontrol Efficacy of B. velezensis ATR2 against Ginger Rhizome Rot Disease

**Figure 6:**
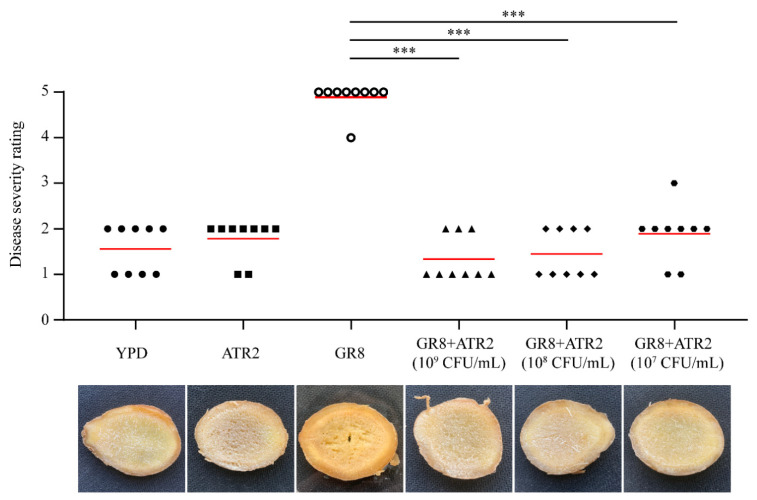
Biocontrol efficacy of *B. velezensis* ATR2 against ginger rhizome rot disease on ginger rhizome slices. The disease severity rating values were calculated using the unpaired two-tailed *t*-test with the GraphPad software (GraphPad Prism 8.4.0, San Diego, CA, USA) (*** *p* < 0.001). The marks represented the disease severity rating values of 9 inoculated ginger rhizome slices. The red lines in the plot area represented the average disease severity rating values.

Given that *B. velezensis* ATR2 exhibited strong antagonistic activity against *B. pumilus* GR8, biocontrol assays on ginger rhizome slices were performed to further evaluate its efficacy against ginger rhizome rot disease. As shown in Figure 6, the slices inoculated with *B. pumilus* GR8 were extensively rotten within 2 days. By contrast, the symptoms of discoloration and tissue breakdown were obviously reduced on the slices inoculated with a mixture of GR8 and ATR2 (10^9^ CFU/mL), even when the bacterial concentration of *B. velezensis* ATR2 was diluted 10-fold (10^8^ CFU/mL) and 100-fold (10^7^ CFU/mL). Additionally, the result of sole inoculation with *B. velezensis* ATR2 on slices, similar to the treatment of YPD liquid medium, proved that *B. velezensis* ATR2 was harmless to the host plant and safe for further use as a biocontrol agent.

## 4. Discussion

Agriculture is the foundation of a country, and the stability and development of agriculture determine the economic level of the country. However, plant diseases and insect pests have always been restricting agricultural development. Currently, control of plant diseases and insect pests mainly depends on agricultural chemicals, whereas long-term and massive use of chemicals has already caused many problems, including drug resistance, environmental contamination, and health hazards [78]. To control plant diseases and insect pests, the demand for safe and eco-friendly alternative products is increasing. In the present study, we characterized the genomic, antimicrobial, and insecticidal properties of a potential biocontrol strain, ATR2. Meanwhile, we identified the antagonistic compound from strain ATR2 against the pathogen of ginger rhizome rot disease and analyzed the biocontrol efficacy of strain ATR2 against ginger rhizome rot disease.

According to the cell and colony morphology, physiological and biochemical characteristics, phylogenetic taxonomy and ANI values analysis, strain ATR2 was finally identified as a member of *B. velezensis*. Genome analysis showed that *B. velezensis* ATR2 possessed a great capacity for synthesizing kinds of secondary metabolites, including three polyketides (macrolactin, bacillaene, and difficidin), three cyclic lipopeptides (surfactin, bacillomycin, and fengycin), one dipeptide (bacilysin), one siderophore (bacillibactin), one bacteriocin (amylocyclicin), one modified peptide (plantazolicin), one putative lipopeptide, and one putative phosphonate. In total, more than 9.25% of the ATR2 genome was devoted to synthesizing secondary metabolites, whereas the genomes of some closely related members of the *B. subtilis* group dedicated only approximately 5% of their capability in production of secondary metabolites [29].

Macrolactin, an efficient peptide deformylase inhibitor, was found in both *B. subtilis* and *B. amyloliquefaciens* [79,80]. Notably, macrolactin A, 7-*O*-malonyl macrolactin A and 7-*O*-succinyl macrolactin A all exhibited antibacterial activities against methicillin-resistant *Staphylococcus aureus* and vancomycin-resistant enterococci [81,82]. Another two polyketides, bacillaene and difficidin, inhibited the biosynthesis of prokaryotic protein [83,84]. Surfactin, a cyclic heptapeptide, was named for its powerful surface-activity [85] and exerted antibacterial, antiviral, and hemolytic performances by altering membrane integrity [86,87]. Both bacillomycin and fengycin exhibited a high fungitoxicity for their membrane permeabilization properties [88,89,90]. In addition to direct suppression against the phytopathogens, the cyclic lipopeptides surfactin and fengycin played a role in eliciting the ISR phenomenon in plants [91]. Bacilysin, a dipeptide antibiotic composed of a nonproteinogenic L-anticapsin at the C-terminus and an L-alanine residue at the N-terminus, exhibited antagonistic activities against kinds of bacteria, *Candida albicans*, and the yeast [92,93,94]. The iron-siderophore bacillibactin participated in specific transport system of taking up and accumulating iron ions from the environment under iron limitation [95]. These nonribosomal peptides and polyketides were synthetized by modularly organized enzymes and all depended on a functional 4′-phosphopantetheine transferase (Sfp), except for the dipeptide bacilysin [96,97]. Amylocyclicin was a circular bacteriocin, which was ribosomally synthesized and showed distinct antibacterial activity against some Gram-positive bacteria [98]. Plantazolicin was a bioactive peptide with thiazole and oxazole heterocycles, which was posttranslationally modified and exhibited antibacterial activity against closely related Gram-positive bacteria [99].

In addition to the ten known bioactive metabolites, one putative lipopeptide and one putative phosphonate predicted in the ATR2 genome were hitherto unknown. According to the previous reports, some organisms produced kinds of phosphonates with antiviral, antibacterial, herbicidal, and antiparasitic properties to provide a competitive advantage [100,101,102]. The lipopeptides, as extensively studied bioactive metabolites, have been related to the biocontrol of multiple plant diseases [51,103,104]. Meanwhile, a series of genes closely related to promoting plant growth and triggering plant immunity were also found in ATR2 genome. These findings suggested that *B. velezensis* ATR2 should be an excellent biological control agent and an important microorganism resource for further exploitation of novel bioactive metabolites.

According to our results, the extracellular metabolites from *B. velezensis* ATR2 exhibited distinct antagonistic activities against multiple plant pathogenic bacteria, plant pathogenic fungi, human pathogenic bacteria, and human pathogenic fungus. Aphids, as widely distributed agricultural piercing-sucking mouthpart pests, mainly damaged the leaves, stems and flowers of vegetables, trees, and flowers. The current control measures for aphids mainly rely on chemical pesticides. The extracellular metabolites from *B. velezensis* ATR2 were also proven to be efficacious in control of aphids in laboratory bioassays and in the field, where corrected mortalities of multiple aphids were almost up to 90%. Collectively, we recommend applying *B. velezensis* ATR2 in agricultural and medical fields, based on its outstanding potential.

The antagonistic compound from *B. velezensis* ATR2 against the pathogen of ginger rhizome rot disease was purified by acid precipitate, preparative thin layer chromatography, and semi-preparative HPLC, and identified as bacillomycin D by LC–ESI–MS/MS analysis. Bacillomycin D, a cyclic lipopeptide from the iturins family, was known to have considerable antifungal activities and effectively inhibit various fungi growth, e.g., *Aspergillus flavus*, *Malassezia globosa*, and *Fusarium graminearum* [51,105,106]. However, the research on the antibacterial activities of bacillomycin D was little reported. According to previous reports, bacillomycin D effectively changed cell membrane permeability, disrupted the cell membrane, advanced the release of cellular contents, and thus induced cell death by reducing the content of ergosterol. Ergosterol was the major sterol moiety which inserted into phospholipids for stabilizing the fungal cell membrane structure [105]. Although bacillomycin D could disrupt the fungal cell membrane, Gong et al. reported that the prime action site for bacillomycin D should be cell wall because the cell walls of mycelia and spores were seriously damaged and deformed in the presence of bacillomycin D [51]. Further work to elucidate the antibacterial mechanism of bacillomycin D against *B. pumilus* GR8 is currently in progress in our laboratory.

Ginger rhizome rot disease, caused by *B. pumilus* GR8, was a soil-borne bacterial disease which gained access via wounds and caused severe rot of ginger rhizomes. In recent years, ginger rhizome rot disease has seriously broken out and threatened ginger production [12]. According to our results, *B. velezensis* ATR2 exerted excellent biocontrol efficacy against ginger rhizome rot disease on ginger rhizome slices. Moreover, *B. velezensis* ATR2 displayed strong bacteriostatic activity against *Ralstonia solanacearum* (Smith) which was the pathogen of bacterial wilt. Bacterial wilt was one of the significant and destructive diseases of ginger [8,107]. In addition, *B. velezensis* ATR2 also showed antibacterial activity against *Enterobacter cloacae* which could cause rhizome rot of edible ginger [53,108]. Based on these results, we propose that *B. velezensis* ATR2 have an excellent potential to control multiple soil-borne bacterial diseases of ginger and could effectively improve food safety by reducing the use of agricultural chemicals.

## Figures and Tables

**Table 1:** Genes related to plant growth promotion in *B. velezensis* ATR2 genome.

Gene and Gene Cluster	Position	Product	Description
*phyC*	2,149,490–2,150,641	3-Phytase	Phytase synthesis
*trpC*	2,265,499–2,266,251	Indole-3-glycerol phosphate synthase	IAA synthesis
*bdhA*	660,137–661,177	Butanediol dehydrogenase	2,3-butanediol synthesis
*ilvN*	2,764,326–2,764,844	Acetolactate synthase small subunit	3-hydroxy-2-butanone synthesis
*ilvB*	2,764,841–2,766,565	Acetolactate synthase large subunit	
*alsD*	3,559,880–3,560,647	alpha-acetolactate decarboxylase	
*alsS*	3,560,708–3,562,420	Acetolactate synthase	
*treP*	820,991–822,406	Trehalose permease IIC protein	Trehalose synthesis
*treR*	824,189–824,905	Trehalose operon repressor	
*speE*	3,685,891–3,686,721	Spermidine synthase	Spermidine synthesis
*speB*	3,684,960–3,685,832	Agmatinase	
*dhbABCEF*	3,110,521–3,129,932	Bacillibactin	Iron transport and utilization
*nirD*	351,031–351,351	Nitrite reductase small subunit	Nitrogen transport and utilization
*nasD*	351,372–353,789	Nitrite reductase large subunit	
*nasC*	353,909–356,041	Assimilatory nitrate reductase catalytic subunit	
*narI*	3,668,425–3,669,096	Nitrate reductase subunit gamma	
*narJ*	3,669,093–3,669,650	Nitrate reductase molybdenum cofactor assembly chaperone	
*narH*	3,669,676–3,671,139	Nitrate reductase subunit beta	
*narG*	3,671,129–3,674,815	Nitrate reductase subunit alpha	
*khtT*	1,027,931–1,028,428	Potassium: proton antiporter subunit KhtT	Potassium transport and utilization
*ktrC*	1,448,732–1,449,397	Ktr system potassium transporter KtrC	
*ktrA*	3,028,064–3,028,732	Potassium uptake protein KtrA	
*corA*	847,676–848,635	Magnesium and cobalt transport protein CorA	Magnesium transport and utilization
*mgtE*	1,338,402–1,339,757	Magnesium transporter MgtE	

**Table 2:** Predicted secondary metabolite clusters in genomes of *B. velezensis* ATR2 and related representative *Bacillus* strains.

Metabolite	Genes and Gene Clusters	Genbank Accession	Main Function	Strains (Genetic Similarity)			
				*B. velezensis* ATR2	*B. velezensis* FZB42	*B. amyloliquefaciens* DSM 7^T^	*B. subtilis* 168^T^
Sfp-dependent nonribosomal synthesis							
Surfactin	*srfABCD*	AJ575642.1	Antibacterial, ISR	82%	91%	82%	82%
Bacillomycin	*bmyABCD*	CP000560.1	Antifungal	100%	100%	93%	ND
Fengycin	*fenABCDE*	CP000560.1	Antifungal, ISR	100%	100%	ND	100%
Bacillibactin	*dhbABCEF*	AL009126.3	Siderophore	100%	100%	100%	100%
Bacillaene	*baeBCDEGHIJLMNRS*	AJ634060.2	Antibacterial	100%	100%	100%	100%
Macrolactin	*pks2ABCDEFGHI*	AJ634061.2	Antibacterial	100%	100%	ND	ND
Difficidin	*difABCDEFGHIJKLMNO*	AJ634062.2	Antibacterial	100%	100%	ND	ND
Sfp-independent nonribosomal synthesis							
Bacilysin	*bacABCDE, ywfAG*	CP000560.1	Antimicrobial	100%	100%	100%	100%
Ribosomal synthesis of bacteriocins and modified peptides							
Plantazolicin	*ptnABCDEFGHIJKL*	FN668567.1	Antibacterial	91%	91%	ND	ND
Amylocyclicin	*acnABCDEF*	CP000560.1	Antibacterial	98%	100%	93%	ND
Unknown metabolite							
Putative lipopeptide	/	/	Unknown	D ^a^	ND	ND	ND
Putative phosphonate	/	/	Unknown	D	ND	ND	ND

^a^ D and ND indicate “detected” and “not detected,” respectively.

**Table 3:** Antagonistic activities of extracellular metabolites from *B. velezensis* ATR2 against some pathogenic microorganisms.

Microorganism	Description	Source or Reference	Diameter of Inhibition Zone (mm) ^a^	Inhibition Ratio (%)
*Bacillus pumilus* GR8	Pathogen of ginger rhizome rot	[12]	27 ± 0.67	/
*Ralstonia solanacearum* (Smith)	Pathogen of tomato bacterial wilt	[59]	33.89 ± 0.19	/
*Xanthomonas campestris* pv. *campestris*	Pathogen of crucifers black rot	[60]	35.11 ± 0.19	/
*Enterobacter cloacae*	Pathogen of rhizome rot of ginger	[53]	19 ± 0	/
*Yersinia pseudotuberculosis* YpⅢ	Pathogen of gastroenteritis	[61]	24.78 ± 0.38	/
*Bacillus anthracis*	Pathogen of anthrax	[62]	20 ± 0.67	/
*Escherichia coli* EHEC O157:H7	Pathogen of diarrhea	[63]	20 ± 0	/
*Klebsiella pneumoniae*	Pathogen of liver abscess	[64]	16 ± 0	/
*Shigella dysenteriae*	Pathogen of bacillary dysentery	[65]	20.56 ± 0.51	/
*Staphylococcus aureus* subsp. *aureus*	Pathogen of cutaneous abscess	[66]	15.11 ± 0.19	/
*Salmonella* Choleraesuis	Pathogen of swine paratyphoid	[67]	17.78 ± 0.38	/
*Salmonella* Typhimurium	Pathogen of enteritis	[68]	16.33 ± 0.33	/
*Acinetobacter baumannii*	Pathogen of bloodstream infection	[69]	14.89 ± 0.19	/
*Fusarium graminearum* Sehw	Pathogen of wheat head scab	[70]	/	75.42 ± 0.72
*Botrytis cinerea*	Pathogen of gray mold	[71]	/	80.83 ± 0.72
*Ceratocystis paradoxa* (Dode) Moreau	Pathogen of pineapple disease of sugarcane	[72]	/	91.67 ± 0.72
*Colletotrichum higginsianum* Sacc	Pathogen of crucifer anthracnose disease	[73]	/	81.67 ± 0.72
*Microsporum gypseum*	Pathogen of tinea corporis	[74]	/	52.5 ± 1.25

^a^ Means and standard deviations were statistically calculated using the software package SPSS v18.0.

## Supplementary Materials

The following supporting information can be downloaded at: https://www.mdpi.com/article/10.3390/microorganisms10010063/s1, Table S1: Physiological and biochemical characteristics of strain ATR2. Figure S1: Bacterial cell and colony morphology of strain ATR2. Figure S2: Phylogenetic tree constructed with 16S rRNA sequences using the neighbor-joining method and bootstrap analysis (1000 replicates) in MEGA. Figure S3: Genomic comparison of the *B. velezensis* ATR2 against closely related *Bacillus* strains. Figure S4: HPLC analysis of the purified antagonistic compound from *B. velezensis* ATR2 against *B. pumilus* GR8.
