# Supplementary material for: Genomic, Antimicrobial, and Aphicidal Traits of Bacillus velezensis ATR2, and Its Biocontrol Potential against Ginger Rhizome Rot Disease Caused by Bacillus pumilus
Source: Microorganisms. 2021 Dec 29;10(1):63. doi: 10.3390/microorganisms10010063 (PMC8778260; doi:10.3390/microorganisms10010063)
Supplement: Supplementary file 1 [file microorganisms-10-00063-s001.zip › microorganisms-1497382-supplementary.pdf]

microorganisms

MDPI

Article

Genomic, Antimicrobial and Aphicidal Traits of *Bacillus velezensis* ATR2, and Its Biocontrol Potential against Ginger Rhizome Rot Disease Caused by *Bacillus pumilus*

Leiqin Liang [1,2], Yajuan Fu [1,2], Sangsang Deng [1,2], Yan Wu [1] and Meiying Gao [1,*]

1 Wuhan Institute of Virology, Chinese Academy of Sciences, Wuhan, 430071, P. R. China; wuyan81@wh.iov.cn; mygao@wh.iov.cn
2 University of Chinese Academy of Sciences, Beijing, 100039, P. R. China; liangleiqin@163.com; fuyajuann@163.com; dengsangsang@163.com
* Correspondence: mygao@wh.iov.cn

Citation: Liang, L.; Fu, Y.; Deng, S.; Wu, Y.; Gao, M. Genomic, Antimicrobial, and Aphicidal Traits of *Bacillus velezensis* ATR2, and Its Biocontrol Potential against Ginger Rhizome Rot Disease Caused by *Bacillus pumilus*. *Microorganisms* **2022**, *10*, x. https://doi.org/10.3390/microorganisms10010063

Academic Editors: Annamaria Bevivino and Maria Maddalena Del Gallo

Received: 22 November 2021
Accepted: 23 December 2021
Published: 29 December 2021

Publisher's Note: MDPI stays neutral with regard to jurisdictional claims in published maps and institutional affiliations.

Copyright: © 2021 by the authors. Licensee MDPI, Basel, Switzerland. This article is an open access article distributed under the terms and conditions of the Creative Commons Attribution (CC BY) license (https://creativecommons.org/licenses/by/4.0/).

Table S1. Physiological and biochemical characteristics of strain ATR2.

Characteristic	Strain ATR2
Pigmentation	Opaque
Anaerobic growth	-[a]
Oxidase	+
Hydrolysis of starch	+
Acid production from:	
D-Arabinose[b]	-
L-Arabinose	+
D-Ribose	+
D-Xylose	+
L-Xylose	-
D-Adonitol	-
D-Galactose	-
L-Sorbose	-
L-Rhamnose	-
Dulcitol	-
Sorbitol	+
Methyl-α-D-glucoside	+
N-Acetylglucosamine	-
Amygdalin	-
D-Lactose	+
D-Melibiose	+
D-Trehalose	+
Inulin	-
D-Raffinose	+
Glycogen	+
Xylitol	-

D-Gentiobiose	-
D-Turanose	+
D-Lyxose	-
D-Tagatose	-
D-Fucose	-
L-Fucose	-
D-Arabitol	-
L-Arabitol	-

[a] + and – represented positive reaction and negative reaction, respectively.
[b] Data were obtained using API 50 CHB test strips.

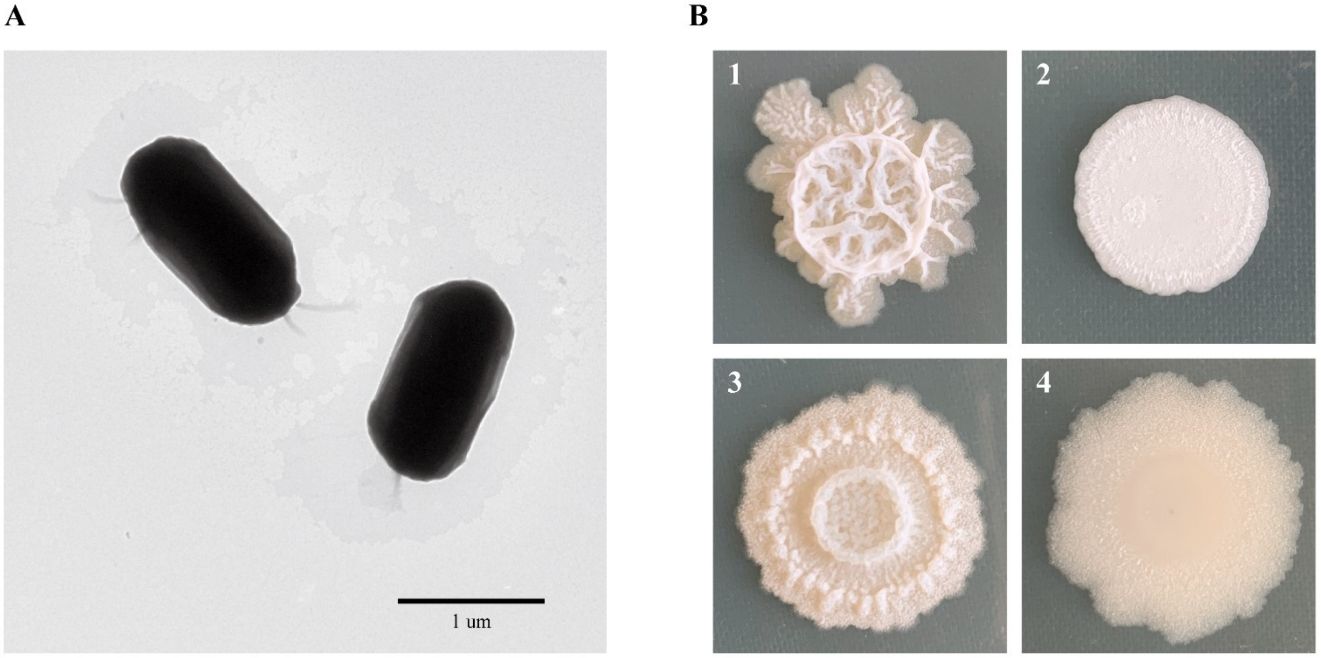

Figure S1. Bacterial cell and colony morphology of strain ATR2. A, The cell morphology of strain ATR2 under transmission electron microscope (TEM). B, The colony morphology of strain ATR2 (1), *B. velezensis* FZB42 (2), *B. amyloliquefaciens* DSM 7^T (3) and *B. subtilis* 168^T (4).

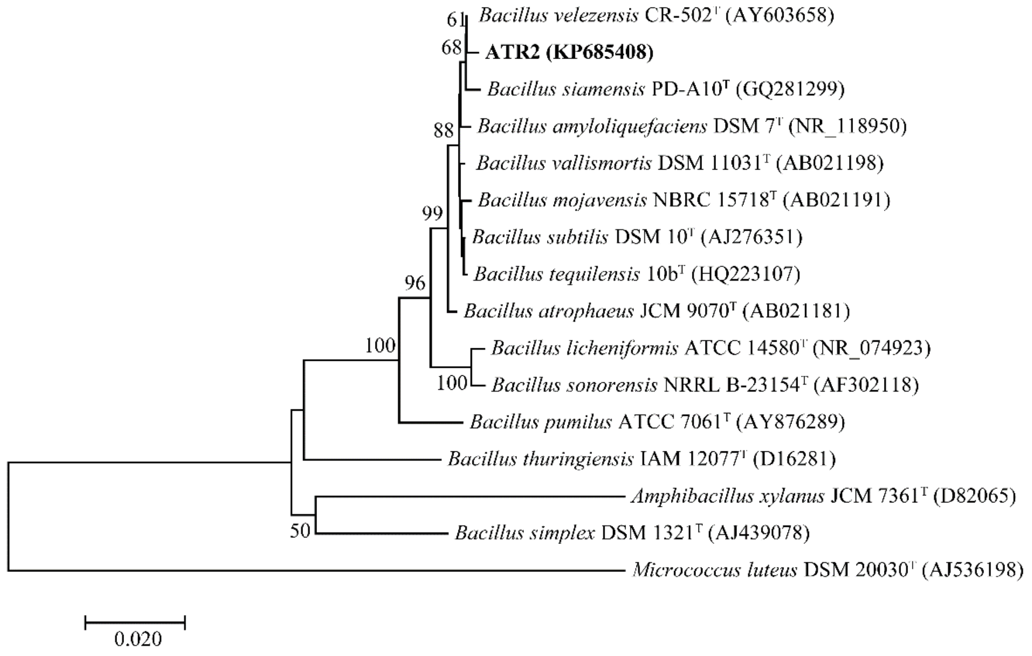

Figure S2. Phylogenetic tree constructed with 16S rRNA sequences using the neighbor-joining method and bootstrap analysis (1,000 replicates) in MEGA. Strain ATR2 was highlighted in bold. Numbers in parentheses represented the GenBank accession numbers. Branch support with value of 50 or more was indicated at the branch nodes. The sequence from *Micrococcus luteus* DSM20030^{T} (AJ536198) was used as the outgroup. The scale bar represented 0.02 substitutions per nucleotide position.

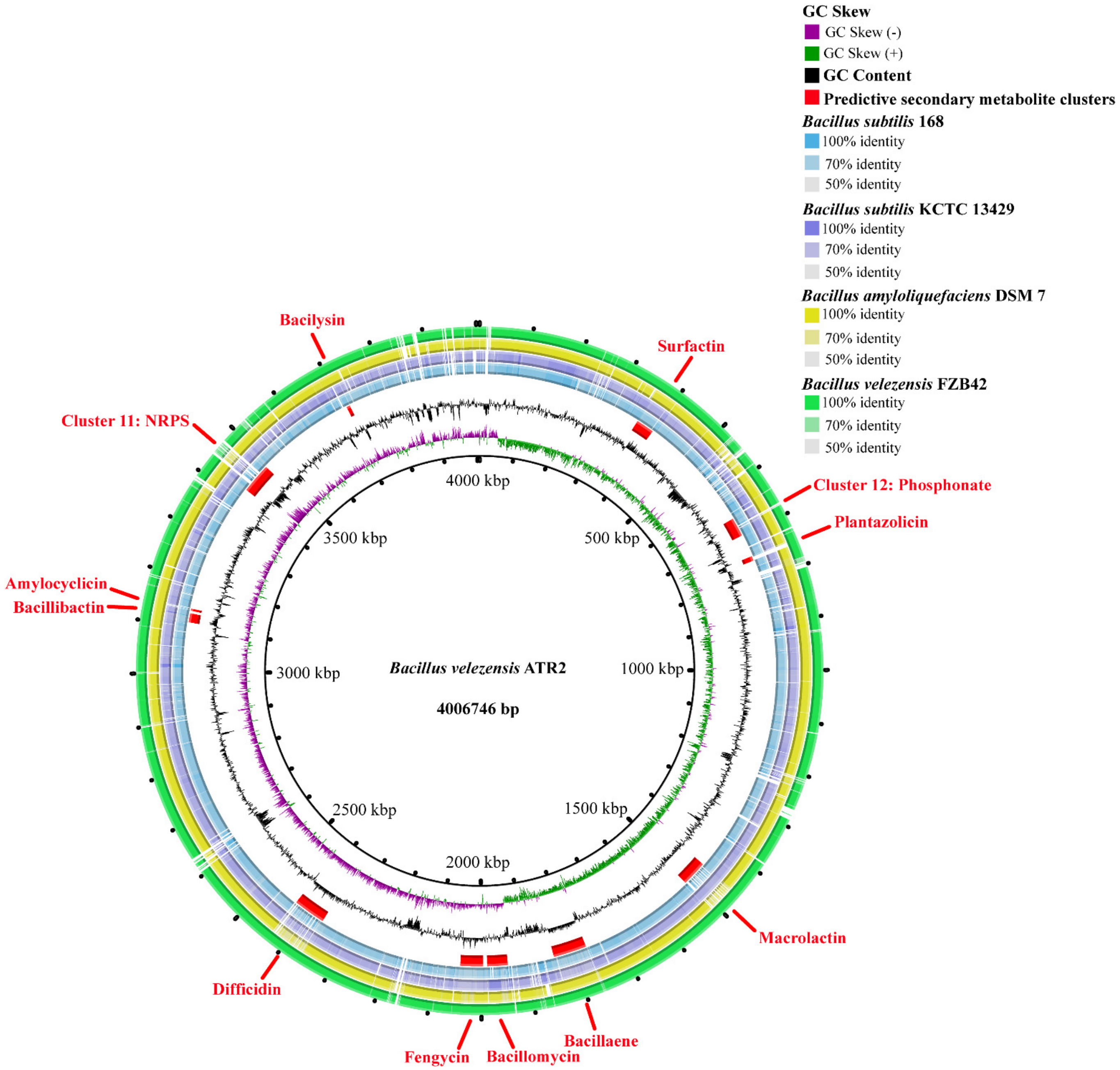

Figure S3. Genomic comparison of the *B. velezensis* ATR2 against closely related *Bacillus* strains. Circles displayed from the inner to outside: (1) GC Skew; (2) GC Content; (3) Predictive secondary metabolite clusters; (4, 5, 6, 7) BLAST comparison of *B. velezensis* ATR2 genome with *Bacillus* strains, *B. subtilis* 168^{T}, *B. subtilis* KCTC 13429^{T}, *B. amyloliquefaciens* DSM7^{T} and *B. velezensis* FZB42, respectively.

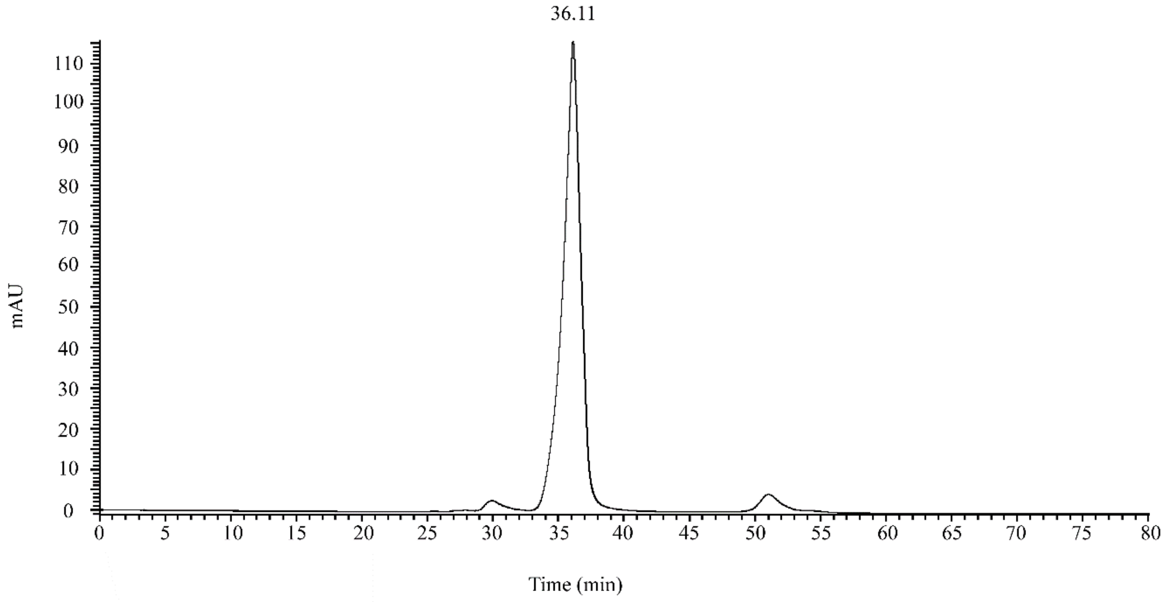

Figure S4. HPLC analysis of the purified antagonistic compound from *B. velezensis* ATR2 against *B. pumilus* GR8.
